# Comparative analysis of innovative behavior in migrant workers and local employees in the Greater Bay Area of China

**DOI:** 10.3389/fpsyg.2025.1576470

**Published:** 2025-03-28

**Authors:** Lanxia Wang, Yiyan Wang, Shuaifang Liu, Hui Zhang, Na Wei, Bilei Zhou, Jun (Justin) Li

**Affiliations:** ^1^School of Business, Shandong Xiehe University, Jinan, China; ^2^Higher Education Mega Center, School of Tourism Management, South China Normal University, Guangzhou, China; ^3^Faculty of International Tourism and Management, City University of Macau, Taipa, Macao

**Keywords:** foreign workers, Innovative work behavior, job autonomy, job performance, leader-member exchange

## Abstract

**Introduction:**

The influx of foreign workers into Chinese cosmopolitan hubs has reshaped workplace dynamics, yet research on their innovation behaviors remains limited, particularly in the post-COVID-19 context. This study examines and compares the relationships between leader–member exchange (LMX), job autonomy, innovative work behavior, and job performance among foreign workers and local employees, addressing gaps in understanding how these factors influence organizational success during and after the pandemic.

**Methods:**

Data were collected from 449 employees (295 foreign workers and 154 local employees) in China’s food and beverage (F&B) industry during the COVID-19 pandemic. A multiple group analysis approach was employed to test hypotheses and compare interrelationships between variables across the two groups. Structural equation modeling (SEM) was utilized to assess direct and indirect effects.

**Results:**

LMX and job autonomy positively correlated with innovative work behavior and job performance for both groups. However, significant differences emerged: job autonomy had a stronger impact on innovative behaviors among foreign workers than local employees. No notable differences were found in LMX effects. These findings highlight the role of cultural and contextual factors in shaping autonomy’s influence on innovation.

**Discussion:**

The study underscores the importance of fostering LMX and job autonomy to drive innovation and performance, particularly as organizations adapt to post-pandemic recovery. For multinational workforces, tailored strategies that address cultural differences in autonomy perception are critical. Practical implications include cultivating inclusive innovation cultures and leveraging autonomy to enhance foreign workers’ contributions. Future research should explore longitudinal impacts of workplace dynamics in diverse sectors.

## 1 Introduction

The food and beverage industry has witnessed a healthy growth over the past couple of years. However, the coronavirus disease 2019 (COVID-19) pandemic deeply affected the field of labour and employment relations (Kutty et al., 2024). Food and beverage industry has been heavily impacted by the repeated lockdowns and social distancing practices (Dedeoğlu et al., 2022; Jaworski, 2021). For example, during the pandemic, many small, family-owned restaurants in countries like India or Brazil stuck to traditional methods, such as dine-in services. Without adopting new strategies like delivery apps or online ordering systems, these businesses struggled to match the agility of larger chains. This gap left them vulnerable to competitors who quickly embraced innovation to survive lockdowns and shifting customer demands. The food and beverage industry offers a critical context for studying innovation. COVID-19 disrupted this sector more severely than many others. Lockdowns forced sudden shifts from dine-in to delivery models. Supply chain breakdowns required rapid adaptation, like sourcing local ingredients. These pressures created a ‘laboratory’ for observing survival-driven innovation. For example, small restaurants in Vietnam pivoted to ghost kitchens, while cafés in Brazil adopted QR code menus overnight. Such examples show how crisis accelerates experimentation. This industry’s reliance on perishable goods and direct customer interaction also makes innovation riskier but more urgent. These factors make it a distinctive setting to explore how innovation behavior unfolds under real-world constraints. The food and beverage industry should explore alternative administrative structures and businesses to improve its global competitiveness (Fraj et al., 2015; Gómez-Rico et al., 2021). Thus, innovation and business transformation could be a critical component of a successful company when we recover a pre-pandemic normal.

Global companies, such as Google, have fostered continuous innovation by smartly managing their employees. Creative employees are normally more productive and instrumental to task performance through innovation (Ji et al., 2019). However, the global economy is heading into recession, tourism growth is slowing, and the competitive struggle among hospitality enterprises is intensifying. It is thus particularly important to encourage employees’ creativity and innovation in the tourism and hospitality industry. More and more restaurants, cafeterias, cafés, fast-food joints, pubs, delis, food manufacturing operations have begun creatively encouraging diverse employees to suggest new ideas that could improve the quality of services or products and further promote sustainable growth and task performance.

Innovation is indeed a crucial factor for the creation and maintenance of sustainable competitive advantage for businesses across various industries. Sustainable competitive advantage refers to a lasting, distinctive edge that a company has over its competitors, and innovation plays a central role in achieving and sustaining this advantage. Among both industry and academia, innovation is a prevalent issue because of how it improves the effectiveness of an organization and other work outcomes (Rodríguez-Victoria et al., 2017). Innovation behaviors and individual performance are closely linked and play a crucial role in the success and competitiveness of individuals and organizations. Innovation behaviors and individual performance play a crucial role in the food and beverage industry, as in any other industry. The food and beverage industry is highly competitive, and companies constantly strive to create new products, improve processes, and meet changing consumer preferences. For example, innovation in product development, such as creating unique and novel flavors, textures, and packaging, is essential for standing out in the market. Individuals who contribute to product innovation enhance the competitiveness of their companies.

We focus primarily on the antecedents of employees generating innovative ideas for development that eventually lead to higher task performance. In extant studies, researchers have often attempted to consider the innovation behavior of employees based on personality type and the influence of personal initiative, such as autonomy. Similarly, other studies focus on situational factors such as leadership style. The literature also identifies a wide range of work-related factors as antecedent factors. For example, innovative employees tend to be more autonomously motivated and happy with increased leader–member exchange (LMX) and job autonomy, which could lead to more innovation behaviors at work. Innovation behavior describes how employees choose to create, share, or apply new ideas in their roles. For example, this could mean redesigning a workflow to save time or trying new methods to solve problems. Leader-member exchange (LMX) focuses on the trust, respect, and two-way support between managers and their team members. Managers with strong LMX relationships often listen actively, share feedback openly, and advocate for their team’s growth. These ideas form the foundation of our study, helping us explore how workplace dynamics drive innovation. Job autonomy and LMX also play critical roles in motivating innovation behavior and job performance as per extant studies on organizational behavior. In this way, with greater autonomy, employees will exhibit more innovation behaviors in the workplace. A meta-analysis by Hammond et al. (2011) finds that job autonomy—one of job characteristics in their study—is a stronger predictor across all predictors. At the same time, if employees are given the freedom associated with autonomy, their working methods become optimal, they develop new job skills throughout their work, and they strengthen task performance. In extant research dealing with the influences on employee service innovation behaviors, individual employee and external characteristics of workplaces are two types of influential factors. Among the many observable external variables that influence employees’ innovation behaviors, LMX is one of most often discussed variables. Within multi-disciplinary research, scholars also suggest that LMX influences organizational behavior—which includes innovation behavior and task performance. However, there exist few exceptional studies on the effect of job autonomy and LMX on employee innovation behavior and task performance in the food and beverage industry. Thus, the first objective of our research is to gather more direct evidence of the degree of the impact of job autonomy and LMX on employee innovation behavior and task performance within the hospitality industry.

Consider the role of national culture on leadership—the level of leadership is associated with innovation behavior as well. Given its importance, the impact of national culture has attracted the interests of researchers investigating the driving forces of innovation capability at the individual employee level. Extant research suggests that foreign-based employees with different values or ideas on day-to-day work might bring in a global perspective because individuals with different backgrounds have unique problem-solving approaches. Therefore, foreign workers are important sources of innovation; these workers can significantly contribute to the business growth of the organization as well as the hospitality industry. Interacting with individuals who possess different knowledges owing to different cultural, social, and religious backgrounds might lead to mutual encouragement and support among employees seeking to recognize and upgrade connections among different pieces of knowledge (Patrick and Kumar, 2012). Innovation behavior is thus essential if firms and organizations are to recover from the economic downturn and thrive. However, according to our best knowledge, the primary LMX studies focus on a single country or region; only few studies explore how different nationalities might influence innovation behavior. In addition, most research in the realm of this relationship emphasizes on theory, thus indicating the need for more empirical evidence. Specifically, researchers have often discussed the association between foreign workers and innovation, but neglected the different aspects of innovation behavior between foreign and local employees in the Chinese hospitality industry. Therefore, when considering LMX and job autonomy, we must determine whether there is a difference between foreign and local employees in the hospitality industry. Given that the relative importance of foreign workers is increasing, the second objective of this study is to explore the differences in LMX, job autonomy, and innovation behavior of foreign workers compared with local employees.

## 2 Literature review

### 2.1 The relationship between LMX and task performance

LMX is the significance of the supervisor’s major role in shaping an employee’s work attitude and performance (Schyns, 2006). Role and social exchange theories employed within research on LMX suggest that exchange patterns within and among individuals might be governed by various types of norms and rules. For instance, consider reciprocity—a common rule in every culture. As per this rule, every action taken by an individual leads to an expectation that another individual will likely exchange goods of equitable value (Khan et al., 2020). Followers show more initiative with their obligations to “pay back” the leader by working better and harder if the leaders provide a considerate and empowering environment to the followers (Grimsdottir and Edvardsson, 2018). Feelings of personal affection for leaders grow along with growth in effective communication between leaders and followers (Le, 2022); this also guides, or motivate, followers to raise their performance and meet leaders’ expectations (Kim and Koo, 2017).

As such, the above arguments offer some empirical evidence in terms of task performance and when using leaders’ as well as followers’ ratings to measure job performance. LMX can directly measure employees’ task success performance, job satisfaction, and work motivation (Kim et al., 2010). Therefore, it has attracted intense attention within hospitality studies. The meta-analytic evidence presented by Martin et al. (2016) also shows that a positive correlation exists between LMX and job performance. To summarize, the following hypothesis is consistent with the results of the meta-analysis presented in the literature:

**Hypothesis 1**: LMX is positively associated with employees’ job performance.

### 2.2 The relationship between LMX and innovation behavior

Extant research has demonstrated that LMX influences the workplace behavior of employees (Martin et al., 2018). Research argues that followers with higher levels of LMX achieve continuous improvement and innovation as they receive more essential resources and opportunities in future (Gajendran and Joshi, 2012). Thus, LMX is said to shape innovation behavior directly.

First, LMX leads to new ideas and solutions generated by employees. Employees with high LMX quality have better chance of obtaining domain-specific information and seek knowledge and expertise from leaders, thus substantially promoting their work ability (Chen et al., 2012). Leader–member interactions include sharing knowledge and experience that cognitively stimulates other employees. Such interactions encourage more employees to think creatively. Thus, through collaboration, it is easier for employees within high-quality employee–supervisor relationships to convince members of the working group to consider new ideas and offer support based on required needs (Le, 2023). Second, in comparison, with lower-quality LMX members, the ability to access and receive more valuable resources and available information creates powerful and influential members with high LMXs. Consequently, members in the high-LMX group are more likely to be trusted and respected by their colleagues in a work team. High-LMX members who obtain special support from their leaders often implement and promote new approaches with more confidence (Scandura and Pellegrini, 2008).

Wang et al. (2015) further find that a strong positive correlation between LMX and innovative job behavior exists within Chinese organizations. From the perspective of social exchange, Li et al. (2016) argue that the positive affect of LMX might influence service innovation exploration. Kim and Koo (2017) and Alsughayir’s (2017) hospitality studies also demonstrate a significant relationship between employees’ perceived LMX and their innovative work behaviors. High-quality LMX relationships encourage innovation. Trust and open communication create a sense of safety, especially in hybrid teams. But in cultures like China’s, teamwork often matters more than bold ideas. Employees might avoid risks to maintain group harmony. Employees who trust their leaders feel safe to share risky ideas (Riaz, 2024; Shrestha et al., 2024). For example, a chef might propose a new dish if their manager actively listens and provides resources. Supportive leaders also shield employees from criticism, allowing creative experiments to thrive. This safety net turns trust into action, driving innovation. Therefore, we the following hypothesize was formulated:

**Hypothesis 2**: LMX will be positively related to employees’ innovation behavior.

### 2.3 Relationship between job autonomy and innovation behavior

Autonomy is the degree of an individual’s freedom to make independent work decisions. Scholars offer a variety of theoretical explanations for why job autonomy explains innovation behavior. For instance, Hornung and Rousseau (2007) find that autonomy leads to more self-directed and self-motivated employee behaviors. Variables such as personal sense of responsibility and control independent could explain this correlation. These beliefs, which are associated with autonomy, can improve employees’ level of confidence as well as cultivate new and novel behavioral patterns when employees perform a wider range of required roles (Le and Lin, 2023).

Janssen (2005) demonstrates that employees try to produce, facilitate, and implement more innovative ideas and behaviors when they perceive they are more influential. Here, autonomy is a major source of practical learning within the manufacturing industry. Individuals can achieve varieties of knowledge and experience through their interactions, their relationship with the environment, and participating in a wider range of work procedures (Lin and Le, 2019). This, in turn, increases the likelihood of raising more questions and greater innovative performance. Ramamoorthy et al. (2005) and De Spiegelaere et al. (2016) also contend that job-related autonomy can predict employees’ innovation behavior. Similarly, Liu et al. (2011) find that employees who enjoy higher autonomy are more likely to produce novel ideas and behaviors. Job autonomy gives employees space to innovate. Workers who control their tasks can experiment without constant oversight. For instance, a barista with flexible hours might test a new coffee blend during slower shifts. Freedom reduces pressure, making creativity feel manageable rather than risky. This balance turns autonomy into a tool for practical innovation. Job autonomy’s role also shifts across industries. Tech workers thrive with freedom, but hospitality staff see fewer gains, as seen in post-pandemic studies. Based on the characteristics and the self-determination theories highlighting the incentive function of job autonomy, we postulate the following hypothesis in line with extant organizational research:

**Hypothesis 3**: Job autonomy is positively related to employees’ innovative work behavior.

### 2.4 Relationship between job autonomy and task performance

Spector (1986) defines job autonomy as “perceived control”; this widely established concept correlates with numerous other variables such as performance, attitude, and well-being. For instance, Fried et al. (1991) observes a systematic and positive relationship between job autonomy and job performance. Hackman and Oldham’s (1980) seminal study provides further evidence for the claim that employees with more job autonomy will raise their performance. The conceptual framework reveals close linkage between autonomy and empowerment, especially with the choice and meaningfulness dimensions of empowerment, which, in two dimensions, contains self-decision making and meaningful concepts, respectively. Empowerment increases job-related performance and innovation behavior, as per empirical studies. Specifically, choice and meaningfulness—two dimensions of empowerment—are linked to job performance.

According to Gellatly and Irving (2001), job autonomy has a direct positive effect on job performance. Langfred and Moye (2004) further confirm that job autonomy enhances performance because employees become more competent and resourceful when they perform their task. Thus, highly motivated employees tend to exhibit high performance. Hence, we assume that job autonomy directly relates to employees’ job performance.

**Hypothesis 4**: Job autonomy is positively related to employees’ task performance.

### 2.5 Relationship between innovation behavior and task performance

Workplace innovation behavior refers to employees’ intentional generation, promotion, implementation of new ideas at work. A wide range of information is collected and considered by innovative employees to create new ideas and upgrade the level of work. Employees are more willing to learn problem-solving for a wide variety of tasks through novel ideation; ultimately this behavior drives them to reach *peak performance*. Indeed, the willingness to learn is a crucial factor influencing job performance; learning during the finishing process also greatly motivates employees to acquire new skills. Notably, this view is similar to Pine et al.’s (2005) challenge–stressor framework. Their meta-analysis shows that challenge stressors have a positive association with motivation and performance. However, only limited studies identify the positive linkage between employee work innovation behavior and performance, such as Gong et al. (2009) and Aryee et al. (2012). Thus, innovative work behavior has a positive impact on performance in the workplace.

**Hypothesis 5**: Innovation behavior is positively related to task performance.

### 2.6 Foreign vs. local employees in the hospitality industry

Many companies and agencies recruit foreign workers, especially from low-wage countries, to supply the existing workforce in the host country. In some industries and regions, there may be a shortage of local workers with the necessary skills or willingness to perform certain jobs. Employers turn to foreign workers to fill these labor gaps. Hiring foreign workers from low-wage countries can be cost-effective for companies. They may offer lower wage demands compared to local workers, which can help reduce labor costs. Janta et al. (2011), citing Abdullah et al. (2009), states that foreign workers bring with them new values and work ethic; they also have an opportunity to learn the local language. Son et al. (2013) note that foreign workers have specific characteristics, such as different cultures, background, and language, which separate them from locals. They provide a variety of ideas and perspectives.

China’s rapid economic growth and prosperity over the past few decades have indeed created employment opportunities that have attracted a diverse group of foreign workers. China has experienced remarkable economic growth, becoming the world’s second-largest economy. This growth has led to an increase in job opportunities across various industries, including manufacturing, technology, finance, and services, making the country an attractive destination for foreign job seekers. China is now the market epicenter of East, Southeast, and Central Asia. China’s prosperity and growth have made it a compelling destination for individuals seeking career opportunities and cultural experiences, resulting in a dynamic and diverse foreign workforce in the country.

**Figure 1:**
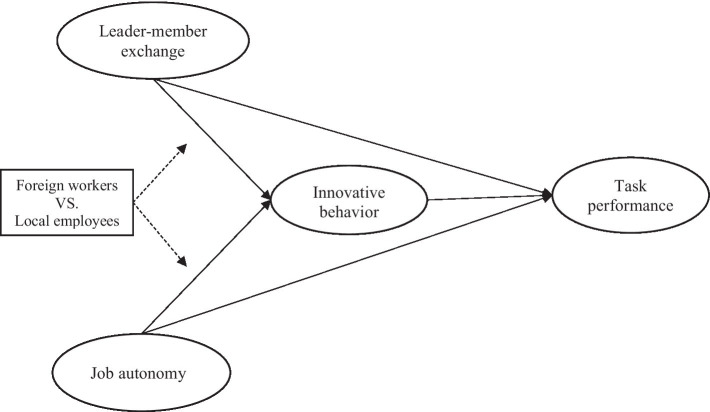
Conceptual framework.

Chinese culture places a strong value on harmony, hierarchy, and the group’s well-being over individual needs and desires. Chinese workplaces tend to have well-defined hierarchies, with clear lines of authority and respect for authority figures. Employees typically follow the directions of their supervisors and managers without questioning them. There is often a cultural emphasis on collectivism and obedience, which can influence employees’ expectations regarding autonomy in the workplace. In terms of employee characteristics, Liu et al. (2011) find that Western employees have a higher need for autonomy than their Eastern counterparts. However, in China, maintaining harmony within the team or organization is highly valued. This emphasis on harmony can sometimes discourage individual employees from asserting themselves or taking actions that might disrupt group cohesion. Pichler et al. (2019) proposes that diversity in nationality at the workgroup level might be related to LMX differentiation, which, in turn, could moderate the relationship between LMX and work outcomes at the individual level. Based on the above, we assume that there are significantly different impacts of LMX and job autonomy on employees’ innovation behavior between foreign workers and local employees. Therefore, the following assumptions are made (Figure 1).

**Hypothesis 6**: The relationship between job autonomy and innovation behavior is significantly different between foreign workers and local employees in China.

**Hypothesis 7**: The relationship between LMX and innovative work behavior is significantly different between foreign workers and local employees in China.

## 3 Methodology

### 3.1 Samples

The Greater Bay Area (GBA) is a dynamic and rapidly developing region in southern China that encompasses several major cities, including Guangzhou, Hong Kong, Macau, Shenzhen, etc. This ambitious initiative aims to transform the GBA into a world-class urban cluster and a leading hub for innovation, technology, finance, and trade. Guangzhou, as one of the key cities within the GBA, plays a crucial role in this regional integration and development. Guangzhou is one of the largest and most economically vibrant cities in China. It serves as a major economic hub within the GBA and plays a pivotal role in driving economic growth and development in the region.

Employees were drawn from 50 large-sized restaurants in Guangzhou, using convenience sampling. To minimize deviations of common method, we use different questionnaires when collecting data of local as well as foreign employees. In the initially stages of the study, the survey procedure and objective were informed clearly to foreign and local employees. We also guaranteed respondents’ anonymity and confidentiality. Back translation ensured the quality and accuracy of the responses. The questionnaire was first translated into Chinese by a translator, and then back translated into English. We distributed 600 questionnaires through mail to participants who were required to evaluate their own LMX relationship and their level of autonomy/independence when performing work tasks. We attempted to increase the response rate by sending regular text reminders; in total, 490 employees (81.73%) responded to the questionnaire. The final sample includes 449 questionnaires (74.83%), after excluding 41 invalid questionnaires.

The study includes more female (55.0%) than male (45.0%) respondents. The highest proportion of respondents were under 30 years of age (37%) at the time of taking the questionnaire; nearly a quarter (24%) were 31 to 39 years old (24%). Almost half the respondents are college-educated, representing 44% of the sample. Over 26% of the respondents are high school graduates and 20% a master’s degree. The respondents include Chinese local employees (35%) and foreign workers (65%). Table 1 reports the respondents’ profile.

**Table 1:** Demographic details of the sample.

Demographic	Frequency	% of respondents
Age		
<31	166	37%
31–39	109	24%
40–49	86	19%
50–59	60	13%
>59	28	6%
Gender		
Male	202	45%
Female	247	55%
Education		
High school graduate	129	29%
Some college/College degree	198	44%
Graduate studies/Graduate degree	88	20%
Others	34	8%
Nationality		
China	154	34%
America	76	17%
German	56	12%
Japan	36	8%
Pakistan	46	10%
South Korea	69	15%
Other	12	3%

### 3.2 Measures

#### 3.2.1 Job autonomy

We measured job autonomy using three items adapted from the Job Diagnostic Survey of Hackman and Oldham (1980). The sample items include “This position will give me the opportunity to use my initiative and make sound decisions using reliable judgment”; “I decide how to start my job”; and “This job increases the opportunities for meeting my needs of independence and personal freedom in the workplace.” The respondents’ answers were assessed on a seven-point Likert scale which ranges from “strongly disagree” to “strongly agree.” The coefficient alpha documented in extant research for this scale is 0.78.

#### 3.2.2 LMX

Four items—affect, loyalty, professional respect, and contribution—were identified to measure LMX (Liden and Maslyn, 1998). The employees rated their LMX using a seven-item scale, with a higher score indicating higher-quality exchanges. The reliability estimate (Cronbach alpha) for the employees’ LMX is 0.87.

#### 3.2.3 Innovation behavior

Innovation behavior was measured by six items adapted from Scott and Bruce (1994). A seven-point Likert-type scale was used to evaluate employees’ innovation behavior in restaurants. The sample items include “Employees create new technology, product philosophies, processes, and techniques” and “Employees promote and share new ideas with others.” The scale of innovation behavior showed the Cronbach’s alpha to be 0.85.

#### 3.2.4 Job performance

Seven items adapted from Williams and Anderson (1991) were used to measure task performance. A seven-point Likert-type scale ranging from 1 (“strongly disagree”) to 7 (“strongly agree”) was used to measure job performance. The sample item includes “The assigned tasks can be done completely and fully by employees.” The scale reliability of task performance is sufficient, with Cronbach’s alpha measuring at 0.79.

### 3.3 Control variables

The extant literature already establishes the control variables (e.g., age, gender, and education) and individual factors indicating significant influence on different groups. We thus include the variables age, gender, and education as controls because they affect employees’ perceptions and work attitudes in China. Wang et al.’s (2015) models help us explore the influencing factors of innovation behavior after controlling for age, gender, and education.

## 4 Results

### 4.1 Descriptive statistics

Table 2 reports the mean, standard deviation, and their mutual correlation for the four factors, namely, LMX, job autonomy, innovative work behavior, and job performance. There is strong positive correlation between LMX and innovation behavior (γ = 0.757, *p* < 0.01); a positive relationship might also exist between LMX and task performance (γ = 0.796, *p* < 0.01). These results provide preliminary evidence for significant correlations between employees’ LMX and their innovation behavior and task performance.

**Table 2:** Means, standard deviations, and correlation.

Variables	Mean	S.D.	(1)	(2)	(3)	(4)
(1) Leader-member exchange	4.18	0.70	1.00			
(2) Job autonomy	4.27	0.62	0.600**	1.00		
(3) Innovative behavior	4.14	0.76	0.757**	0.603**	1.00	
(4) Task performance	4.31	0.65	0.796**	0.623**	0.700**	1.00

*p < 0.05; **p < 0.01; ***p < 0.001.

Both innovation behavior (γ = 0.603, *p* < 0.01) and task performance (γ = 0.623, *p* < 0.01) are significantly associated with employees’ job autonomy. In fact, these positive effects are also verifiable. Finally, innovation behavior correlates positively with task performance (γ = 0.700, *p* < 0.01).

### 4.2 Confirmatory factor analysis

First, we propose four competing alternative models to evaluate which model fits the existing data. Table 3 presents the comparison of the results of these models. We find that Model 1 fits the data well (Model1: *χ*^2^ = 282.76; df = 84; CFI = 0.96; GFI = 0.97; RMSEA = 0.07). For a direct comparison of Model 1, we identify three competing models. A three-factor model is developed by combining LMX and job autonomy into one single factor (Model 2: *χ*^2^ = 1083.10; df = 88; CFI = 0.81; GFI = 0.77; RMSEA = 0.16). Second, a two-factor model is identified by loading the LMX, job autonomy, and innovation behavior into a single factor (Model 3: *χ*^2^ = 1253.64; df = 90; CFI = 0.78; GFI = 0.74; RMSEA = 0.17). Finally, we compare the four-factor model with the global one-dimensional model that combines all factors (Model 4: *χ*^2^ = 1458.33; df = 91; CFI = 0.74; GFI = 0.71; RMSEA = 0.18). The result reveals that the four-factor solution is more optimal. It provides a better fit than the other models do, as previously reported. Thus, the solution is appropriate for the given data.

**Table 3:** Model comparisons.

Models	Factors	χ^2^	df	CFI	GFI	RMSEA	comparison	Δχ^2^	Δ*df*
Model 1	Four factors	282.76	84	0.96	0.93	0.07			
Model 2	Three factors; based on Model 1, LMX and JA were combined into one factor	1083.10	88	0.81	0.77	0.16	2 vs. 1	800.34***	4
Model 3	Two factors; based on model 1, LMX, JA and IB were combined into one factor	1253.64	90	0.78	0.74	0.17	3 vs. 1	170.54***	2
Model 4	One factor; all four factors were combined into one factor.	1458.33	91	0.74	0.70	0.18	4 vs. 1	204.69***	1

*p < 0.05; **p < 0.01; ***p < 0.001.

The measurement model was estimated using AMOS 22.0 to conduct confirmatory factor analysis. The analysis shows the model is well-fitting [*χ*^2^(df.) = 282.756 (84), *p* < 0.00; CFI = 0.962; GFI = 0.927; NFI = 0.947; RMSEA = 0.07; SRMR = 0.034]. The values for Cronbach’s alpha of all the variables are estimated to be greater than 0.70 (see Table 4). That is, the construct reliabilities range between 0.881 (LMX) to 0.909 (task performance). Further, each factor loading value ranges from 0.793 to 0.875 for LMX, 0.731 to 0.917 for job autonomy, 0.776 to 0.864 for innovation behavior, and 0.794 to 0.868 for employee task performance, with *p* < 0.001 significance. Therefore, results provide evidence of convergent validity. Table 4 also shows good discriminant validity, comparing the average variance extracted (AVE) of a construct to the squared correlations between the construct and any other construct. We find that the AVE is higher in this study.

**Table 4:** Results of confirmatory factory analysis.

Paths	Loadings (*t*-value)	CR	AVE	Cronbach’s α
Leader-member exchange		0.881	0.650	0.881
LMX1	0.801***			
LMX2	0.793***(18.681)			
LMX3	0.815***(19.360)			
LMX4	0.815***(19.352)			
Job autonomy		0.888	0.727	0.879
JA1	0.731***			
JA2	0.917***(18.967)			
JA3	0.897***(18.716)			
Innovative behavior		0.894	0.680	0.890
IB1	0.766***			
IB2	0.863***(19.396)			
IB3	0.864***(19.418)			
IB4	0.800***(17.762)			
Task performance		0.91	0.716	0.909
TP1	0.865***			
TP2	0.868***(24.367)			
TP3	0.855***(23.715)			
TP4	0.794***(20.902)			

Fit indices of the reflective measurement model: *χ*^2^(df.) = 282.756 (84), *p* < 0.00 (*χ*^2^/df. = 3.366); CFI = 0.962; GFI = 0.927; NFI = 0.947; RMSEA = 0.07; SRMR = 0.034. *p < 0.05; **p < 0.01; ***p < 0.001. The average variance extracted (AVE) and the composite reliability (CR) appear in the reflective scales to evidence reliability.

### 4.3 Hypotheses testing

**Figure 2:**
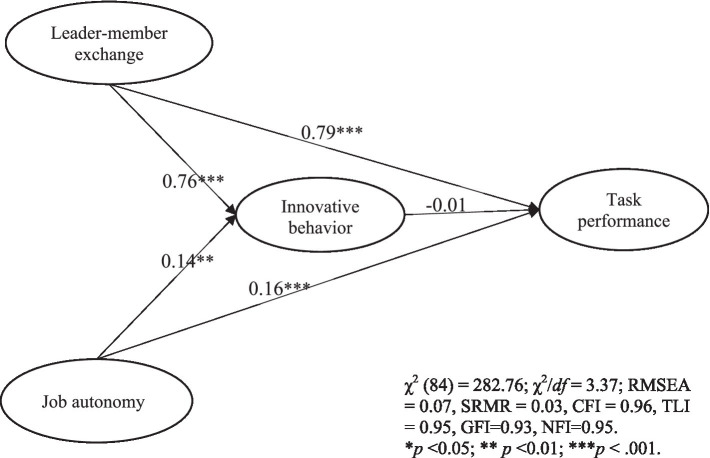
Paths estimate.

This study employs Structural Equation Modeling (SEM) as the analytical framework to examine and assess the interrelationships between variables. Using Structural Equation Modeling (SEM) to test relationships is a common approach in research across various fields. Structural Equation Modeling (SEM) is a statistical technique used in social sciences, psychology, economics, and other fields to analyze the relationships between observed variables and latent variables, as well as to test theoretical models. It is a comprehensive statistical approach that combines factor analysis and regression analysis to test and estimate the relationships among observed and latent variables within a hypothesized model. Hypotheses 1 and 2 suggest that LMX influences job performance and innovative work behavior positively. Hypotheses 3 and 4 assume that job autonomy is positively correlated with innovative work behavior as well as job performance, respectively. Hypothesis 5 predicts that employee innovation behavior will have a positive impact on task performance. As presents in Figure 2, the assumed model fits the observed data well [*χ*^2^(84) = 282.76; *χ*^2^/df = 3.37; RMSEA = 0.07, SRMR = 0.03, CFI = 0.96, TLI = 0.95, GFI = 0.93, NFI = 0.95]. Both the coefficients from LMX to task performance (β = 0.79, *p* < 0.001) and innovation behavior (β = 0.76, *p* < 0.001), as shown in Figure 2, are significant and positive, supporting Hypotheses 1 and 2. The coefficients of job autonomy to innovative work behavior (β = 0.14, *p* < 0.01) and job autonomy to job performance (β = 0.16, *p* < 0.001) are significant and positive, supporting Hypotheses 3 and 4. However, innovative work behavior did not significantly affect job performance, which negates Hypothesis 5.

### 4.4 Results of testing hypotheses 6 and 7

To test hypotheses 6 and 7, the relationships between LMX, job autonomy, and innovative work behavior are expected to be significantly different between foreign workers and local employees. We accordingly employ a multiple group analysis approach by first dividing all 449 respondents into two groups: foreign workers (*n* = 295) and local employees (*n* = 154). To determine if the measurement models across groups are invariant, we conduct testing for measurement invariance before comparing the path coefficients for both groups. Two models (Table 5) are not statistically different [Δ*χ*^2^(19) = 27.06, *p* = 0.10 > 0.01], supporting full-metric invariance. Both non-restricted model and full-metric invariance models show satisfactory fit.

**Table 5:** Measurement invariance test.

Group	Models	*χ* ^2^	df	RMSEA	CFI	NFI	Δ*χ*^2^	Full-metric invariance
	Non-restricted model	576.43	168	0.07	0.93	0.90	Δ*χ*^2^(19) = 7.06 *p* = 0.10 > 0.01 (insignificant)	Supported
	Full-metric invariance	603.49	187	0.07	0.92	0.89		

The nested baseline models apply to AMOS and allow the coefficients to vary by specifying the group. All fit indices rely on a baseline model and indicate that the model is good and fits the data (Table 6). The statistics of the constraint-nested model can be compared with the baseline model by conducting a chi-square difference test to determine if there exist any differences between first timers and repeaters of path coefficients. Table 6 presents the results from checking the significance of the coefficients in the series. The result indicates that the path coefficient of LMX and job autonomy affect innovation behavior between both employee groups based on the chi-squared test. Specifically, the effect of job autonomy for foreign workers (β = 0.19, p < 0.01) on innovation behavior is more positively stronger than for local employees (β = −0.28, *p* > 0.05). However, the effect of LMX on innovation behavior is not significantly different [Δχ^2^(1) = 1.58, *p* = 0.21 > 0.05] between foreign workers (β = 0.69, *p* < 0.001) and local employees (β = 0.86, *p* < 0.001). In both groups, the effect of LMX on innovation behavior is critical: There is no statistically significant difference between foreign workers and local employees. Hence, Hypothesis 6 is supported, whereas Hypothesis 7 is rejected.

**Table 6:** Invariance tests of the structural models for travel arrangement groups.

Paths	Foreign workers		Local workers		Baseline model (freely estimated)	Nested model (constrained to be equal)
	Coefficients	*t* Value	Coefficients	*t* Value		
LMX → IB	0.69***	9.65	0.86***	8.97	*χ*^2^(72) = 576.43	*χ*^2^(71) = 578.01^a^
JA → IB	0.19**	2.98	−0.28	−0.37	*χ*^2^(72) = 576.43	*χ*^2^(71) = 581.20^b^

a Δ*χ*^2^(1) = 1.58 p = 0.21 > 0.05 (insignificant).

b Δ*χ*^2^(1) = 4.77 p = 0.03 < 0.05 (significant).

## 5 Discussion and conclusions

The food and beverage industry is experiencing unprecedented challenges from COVID-19. This study uses data of food and beverage industry employees in China at this challenging time, and then develops and empirically tests an integrated model that includes LMX, job autonomy, innovation behavior, and task performance. Using conceptualization and empirical testing methods, it contributes to the extant literature that analyzes foreign workers and local employees based on the relationships between LMX, job autonomy, and innovation behavior. The results reveal that: (1) Job autonomy affects employees’ innovative work behaviors and job performance. (2) There is no significant relationship between innovation behavior and task performance. (3) There is a significant difference between foreign workers and local employees with respect to the impact of job autonomy on innovative work behaviors. (4) There are no differences in the effects produced by LMX on innovation behaviors for both employee groups. That is, for both groups, LMX is crucial.

### 5.1 Theoretical implications

This study focuses on the impact of COVID 19 and ongoing adaptation of the food and beverage industry, with specific attention to the diversity management and employees’ innovation behavior during this challenging time. First, we explored the critical role of food and beverage industry employees’ LMX and job autonomy on innovative work behavior and outcome. Li et al. (2012) did recognize the potential importance of LMX within the context of hospitality, suggesting further research on the correlation between LMX and employee outcomes. Thus, we proposed a comprehensive model that exemplifies how LMX and job autonomy lead to innovative work behavior and better job performance of food and beverage industry employees. That is, high-quality leader–member relationships, as perceived by employees, ensure effective innovation and higher task performance.

First, the positive relationship between LMX and employee outcomes was explored. The social exchange theory interprets the influence of LMX on innovation behaviors by highlighting how LMX perception could further lead to higher innovation behaviors. In line with previous research findings, this study also supports the view that the motivational role of job autonomy affects innovation behavior and task performance significantly. We thus find a positive relationship between job autonomy and innovation behavior. In line with extant research, we also find that personal performance largely depends on job autonomy. However, Battistelli et al. (2013) reveal that autonomous activities may negatively impact innovative performance because of culture differences. The results herein do not support the view that autonomous activities promote employees’ innovation performance. Within organizational behavior research, there are calls to focus more deeply on the important role of proactive behaviors in work outcomes. In this aspect, our work is valuable.

Second, this study contributes to a sounder theoretical understanding of how the correlation between innovative work behavior and job performance is not significant. Contrary to prior studies (e.g., Kim and Koo, 2017), these findings reveal no direct link between innovation behavior and task performance. This discrepancy may stem from contextual factors. For example, in China’s collectivist culture, employees might prioritize team cohesion over individual innovation, diluting short-term performance gains. Additionally, innovation in the food and beverage industry often involves incremental adjustments (e.g., menu tweaks) rather than disruptive changes. Such minor innovations may not immediately enhance measurable outcomes like efficiency or sales. This suggests that innovation’s impact on performance could depend on cultural norms and innovation type. Importantly, in support, extant research suggests that the participation of employees in innovative processes may lead to fewer job resources being available for task performance, and thus the relationship between innovation behaviors and performance may be smaller or null. Studies also explain how the degree to which subordinates support and oppose creative inputs is affected by the achievement of the organization’s objectives.

Third, this study also explores the impact of a manager’s goal achievement on the outcome of innovation. We find that in scenarios where supervisors have a stronger performance goal orientation, there exist more conflicts between innovators and colleagues and a weaker relationship with job performance. Other factors, such as self-regulation, goal orientation, self-control, working climate, and job characteristics, can further explain this tendency. Thereby, the main model of the current research is different from extant investigations. Thus, we contribute more effectively to the relevant variables of organizational and behavioral studies.

Fourth, previous studies reveal that certain personal characteristics can influence innovation behaviors. For instance, Alsughayir (2017) suggests that future studies could also consider these characteristics. This study compares foreign and local employees in China’s hospitality sector—a rarely studied group. This reveals how cultural differences shape innovation. For example, foreign workers benefit more from job autonomy, while LMX matters equally for both groups. This study challenges assumptions that innovation always boosts performance. In collectivist settings, team harmony may delay measurable gains. These insights extend LMX and Job Demands-Resources theories to multicultural workforces. They also guide managers in tailoring support for diverse teams.

In conclusion, we extend the study of innovation behavior of foreign employees in Chinese restaurants. Such a study helps integrated the larger LMX framework—perhaps the most important theory within organizational behavior—into the Chinese context. Importantly though, both formal and informal institutional arrangements, that is, culture, affect job autonomy. Indeed, differences in individual-level may, to a great extent, stem from the fact that, in different organizations, the degree of autonomy employers give their employees can vary. Our results confirm the findings of extant studies: That is, culture has a significant impact on employees’ job stress experience. The cultural differences between foreign and local employees may influence employees’ perceived job autonomy and their work behavior. Owing to the unique background of this study, a cross-cultural perspective on employees’ innovation behaviors provides critical knowledge. Conceptually, the link found between LMX and innovation behavior could be generalized to other cultural contexts. In many other Asian countries such as China, few extant studies empirically examine these relationships. This is a significant omission, especially given how the dominant cultural belief in China emphasizes interdependence between supervisors and employees. Based on studies that prove the positive effect of LMX on employee innovation behavior, we provide preliminary evidence in support of the link between LMX and innovation behavior.

### 5.2 Managerial implications

This study findings have implications for managers or human resource specialists. First, higher LMX and job autonomy may lead to better work performance. Thus, it is important to design a leadership development program that fits well with the unique corporate culture and needs of employees. Leaders should be encouraged to develop positive relationships with their members in the workplace and employees should realize that building a strong relationship with their supervisors is vital as well. This is especially true in the food and beverage industry, where employees dominate. If these are reciprocal relationships between employees and leaders, employees will deliver the best quality of services. Similarly, we also show that embracing an environment that supports autonomy enhances individual task performance. Thus, creating an environment in which an employee can choose how autonomous he or she wishes to be can inspire other employees and improve performance.

Second, many organizations owe their success and competitiveness to organizational innovation. Thus, understanding the significant predictors of employee innovation behavior is vital. Business leaders can shape a more creative work environment if they follow the results of this study, where the results show that managers should encourage employees who can take ownership of their autonomy at work. Innovation ability can also be cultivated through establishing strong LMX between leaders and their followers; for example, innovation is more likely when leaders of different backgrounds and areas of expertise share their ideas, and offer followers a rational reason to support such ideas. Supervisors should also adjust their style to accommodate the requirements of their subordinates. For example, supervisors should provide a “supportive autonomy” that is appropriate to an individual’s level of capability. Studies reveal that work environments that are more autonomous in nature have not only higher job satisfaction, but also better productivity. Effective businesses management should encourage the development of a positive connection between leaders and individuals as well as amongst coworkers in order to create an atmosphere of open and free communication. Employee empowerment thus gives employees greater responsibility and autonomy. This, in turn, makes them feel more engaged with their tasks, encouraging job and task-level innovation as well.

Third, this study findings underscore how LMX and job autonomy significantly affect the implementation of creative ideas suggested by foreign and local employees. Both types of employees approach LMX in a way that exemplifies the importance of effective communication within a group; its influence on innovation behavior is thus insignificant. From a practical point of view, strong employment relationships create a pleasant atmosphere for both foreign and local employees. A manager must treat his or her foreign and local employees well and create a considerate corporate atmosphere. For example, managers could pursue better work–life balance personally and model appropriate behavior, while supporting employees in similar pursuits. This strategy could build stronger relationships between managers and their employees. On the other hand, employees, from their view, add unique value and perspective. While discussing employee performance goals, managers can create a well-organized employee development plan. Improving the quality of LMX could further cultivate innovation behaviors of employees.

Fourth, by examining the connection between LMX and innovative work behaviors, foreign and Chinese employees can increase innovation behaviors by better understanding each other’s values and social ethics. We also demonstrate that the effect of job autonomy on innovation behavior has significant differences between these two groups: Autonomy can stimulate foreign employees to obtain more innovation opportunities compared with local employees. Thus, organizations must also consider foreign and local employees’ autonomy and the different effects on employee attitudes and work outcomes. One way to familiarize managers with their foreign employees’ wants, needs, and goals is to establish open and honest communication and provide the autonomy for such employees’ innovation behavior. Foreign employees with greater sense of autonomy can independently make decisions; they can be more committed to their jobs within clear boundaries. They have more freedom to accomplish duties and tasks specified in a job description in the modern workplace. Furthermore, they are engaged, enrolled, and empowered to make decisions, share information, and embrace new work experiences.

## 6 Limitations and suggestions for future research

This study has several limitations that warrant further investigation. First, this study design limits insights into how variables evolve over time. Future research should adopt longitudinal methods to track changes in LMX, job autonomy, and innovation behaviors across multiple time points. For example, a three-wave study could assess how shifts in leader-employee relationships predict later changes in innovation outcomes. This approach would clarify causal mechanisms while controlling for baseline differences. Second, while employee perceptions directly shape their behaviors, self-reports may inflate correlations due to common method bias. To address this, future studies could combine self-ratings with objective metrics. For instance, supervisor evaluations of job performance or third-party assessments of innovation outputs (e.g., patents, process improvements) could complement employee surveys. Triangulating data sources would strengthen validity and reduce subjectivity. Third, while we compared foreign and local employees, deeper analysis of collectivism’s role is needed. Future work could measure collectivist values (e.g., using Hofstede’s cultural dimensions) and test their moderating effects. For example, collectivism might weaken the link between job autonomy and innovation for local employees, as group harmony could outweigh individual initiative. Including such analyses would clarify how cultural norms shape innovation dynamics in China’s unique context. Fourth, we consider food and beverage industry employees as research objects in this study; our findings are thus not generalizable to other settings. Therefore, more empirical data should be collected to test the proposed hypotheses.

## Data Availability

The original contributions presented in the study are included in the article/supplementary material, further inquiries can be directed to the corresponding author.
